# Bone Marrow Granulomatosis in Acute Q Fever

**DOI:** 10.7759/cureus.18782

**Published:** 2021-10-14

**Authors:** Joana Azevedo Carvalho, Susana Pereira, Leonor Boavida, Nuno Gião, Ana Bastos Furtado

**Affiliations:** 1 Department of Internal Medicine IV, Hospital Professor Doutor Fernando Fonseca, Amadora, PRT; 2 Department of Hematology, Hospital Santo António dos Capuchos – Centro Hospitalar Lisboa Central, Lisbon, PRT; 3 Department of Pathologic Anatomy, Hospital Santo António dos Capuchos – Centro Hospitalar Lisboa Central, Lisbon, PRT

**Keywords:** fever of unknown origin, fibrin ring granuloma, doughnut granuloma, coxiella burnetii, q fever

## Abstract

Reported cases of Q fever in people living in urban areas after occasional contact with farm animals or infected pets such as dogs and cats have been increasing. The diagnosis of Q fever is usually laborious due to unspecific and variable clinical manifestations. The most common clinical presentation is an influenza-like illness with varying degrees of pneumonia and hepatitis. Acute hepatitis is more frequent than pneumonia in countries where the disease is endemic, such as in Portugal.

We report a case of acute Q fever with hepatic and bone marrow involvement presented as fever of unknown origin (FUO) in a 56-year-old sportive hunter man. Typical fibrin ring granulomas (doughnut granulomas) were found in the bone marrow biopsy and were essential for the diagnosis.

Bone marrow involvement is considered a rare manifestation of Q fever. *Coxiella* infection activates a granulomatous inflammatory response that can lead to persistent immune cell activation. Doughnut granulomas are not pathognomonic but they are highly specific for the diagnosis of Q fever.

## Introduction

There has been an increase in reports of sporadic cases of Q fever in people living in urban areas after occasional contact with farm animals or infected pets such as dogs and cats [1]. *Coxiella burnetii* infection in humans can be acute or chronic and long-term sequelae have been considered as a third category of the disease. Q fever is usually asymptomatic or manifests as a mild disease with spontaneous recovery. The most common clinical presentation is an influenza-like illness with varying degrees of pneumonia and hepatitis. Endocarditis is the most frequent chronic presentation [2].

Patients with hepatic involvement can however present with prolonged “fever of unknown origin” (FUO) associated with elevation of liver enzymes and hepatomegaly without jaundice. Liver biopsy shows granulomatous hepatitis with typical “doughnut” granulomas. This presentation is more frequent than pneumonia in countries where the disease is endemic such as France, Spain, Israel, Taiwan, and Portugal [3,4].

We present a case of a sportive hunter who presented with an acute Q fever with typical hepatic involvement associated with a significant systemic inflammatory response and granulomatous infiltration of the bone marrow.

## Case presentation

A 56-year-old man presented to the emergency department with a two-week history of fever with rigors, night sweats, myalgia, asthenia, and anorexia. The fever had a partial response to acetaminophen one gram every eight hours. The patient had a history of hypertension and diabetes mellitus type II, with optimal control with oral medication. He was a sportive hunter that lived in a Portuguese rural area, had a vaccinated dog, and had no recent travel history. There was no family or sexual history of note.

Physical examination was unremarkable except for a slight increase in heart rate (108 bpm). Laboratory investigation revealed a serum C-reactive protein of 30.36mg/dL without leukocytosis, elevated erythrocyte sedimentation rate (ESR) of 101mm/h, an acute liver injury with normal bilirubin (AST 223U/L, ALT 224U/L, GGT 737U/L, FA 303.01U/L, total bilirubin 1.09mg/dL) with a spontaneous elevation of INR of 1.4 and hypoosmolar hyponatremia (Na 130mmol/L) - (Table 1). Viral serologies including HIV, hepatitis A, B, C, cytomegalovirus, and SARS-CoV-2 were negative. A false positivity for EBV (IgM and IgG VCA) was confirmed by a blood polymerase chain reaction (PCR) assay. Blood and urine cultures were negative. Thoracic radiography showed a diffuse interstitial infiltrate and the thoracoabdominal computed tomography (CT) scan showed diffuse bronchial thickening and hepatosplenomegaly associated with light pleural and peritoneal serositis. Small adenopathies (periaortic and in the hepatic hilus) with the largest diameter of 11mm were also reported.

**Table 1:** Laboratory blood test results *, Not available; AST, aspartate aminotransferase; ALT, alanine aminotransferase; ALP, alkaline phosphatase; LDH, lactate dehydrogenase; GGT, gamma-glutamyl transferase; CPR, C-reactive protein; ESR, erythrocyte sedimentation rate; ACE, angiotensin-converting enzyme

	Admission	One week after admission	Three weeks after admission	One month after admission	Three months after admission
Hemoglobin (g/dL)	11.7	10.2	13.5	12.5	15.3
Hematocrit (%)	34.6	32.4	40.1	38.2	44.9
Leukocytes (x10^9^/L)	9.9	4.9	13.8	14.9	8.7
Neutrophils (x10^9^/L)	7.3	2.9	9.6	9.7	4.1
Platelets (x10^9^/L)	197	101	684	442	201
Sodium (mmol/L)	130	136	136	139	143
AST (U/L)	223	61	24	17	24
ALT (U/L)	224	94	37	19	19
ALP (U/L)	303	210	*	103	88
LDH (U/L)	386	231	141	136	153
GGT(U/L)	737	231	87	65	38
Total bilirubin (mg/dL)	0.68	*	0.20	*	*
Albumin (g/dL)	3.42	2.11	4.16	*	4.51
CPR (mg/dL)	30.4	21.6	8.1	5.8	0.1
ESR (mm/h)	101	100	56	5.8	0.1
Ferritin (ng/mL)	7,662	4,300	*	*	171
ACE (U/L)	*	*	163.7	85.0	*

The patient was admitted to an internal medicine department for further investigation. Empirical antibiotic therapy with IV ceftriaxone was started with no response and the patient maintained fatigue and daily high-grade fever (maximum temperature 39.1ºC).

Multiple infectious causes were excluded by serology and/or urinary antigen tests, such as *Mycoplasma pneumoniae*, *Legionella pneumophila*, *Rickettsia *spp, *Brucella*,* Leptospira, Bartonella, Borrelia burgdorferi Franciella tularensis, Ehrlicia chanffensis* and *Chlamydia pneumoniae*. PCR for *Mycobacterium tuberculosis* in the bronchoalveolar lavage and interferon-gamma release assay was also negative. *C. burnetii* serologies were inconclusive due to the low title of phase I IgM with a negative phase II IgM and IgG. Cardiac vegetations were excluded by transthoracic echocardiogram.

Further blood tests revealed a low title of anti-nuclear antibodies (ANA) (1/160) with a fine speckled pattern, low title of anti-dsDNA, low levels of C4 (3.8mg/dL) with normal levels of C3, elevated serum angiotensin-converting enzyme (ACE) levels of 163.72U/L and high levels of ferritin (7,662ng/mL). No other autoantibodies related to lupus, autoimmune hepatitis, or vasculitis were positive. Thoracic CT scan excluded hilar or mediastinal lymphadenopathies. Prostatic-specific antigen and alpha-fetoprotein were normal.

**Figure 1:**
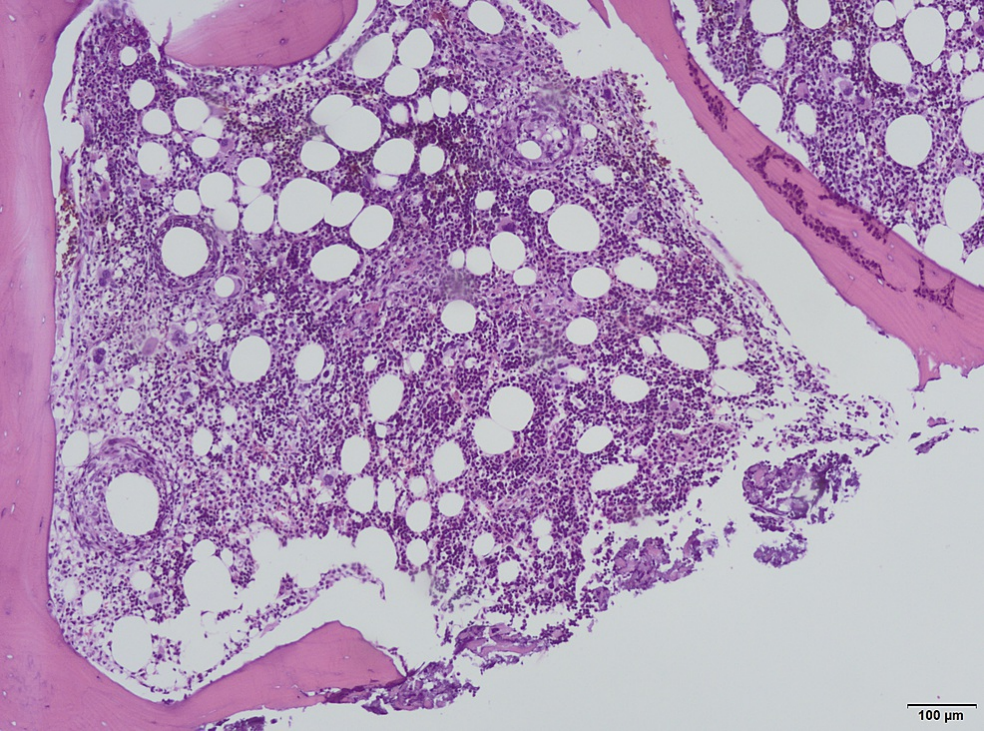
Bone-marrow specimen demonstrating multiple fibrin-ring granulomas (hematoxylin & eosin stain; x100)

**Figure 2:**
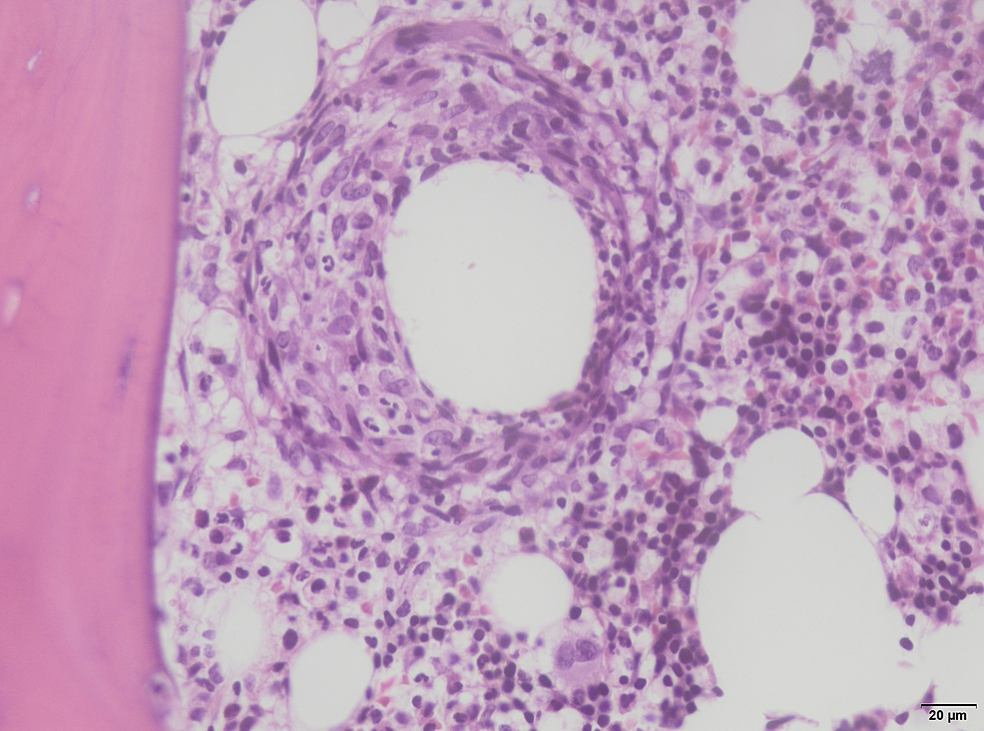
Higher magnification of bone marrow specimen showing non-necrotizing granuloma with a central fatty vacuole and the surrounding macrophages (hematoxylin & eosin stain; x400)

Bone marrow cultures were sterile. Myelogram and bone marrow biopsy revealed a population of 10% of polyclonal CD138+ plasma cells associated with diffuse granulomas with a central lipid vacuole (Figures 1, 2).

Empirical doxycycline was started due to the suspicion of zoonosis. The patient's condition evolved with hypercalcemia (11.5mg/dL) and persistent inflammation with high ESR and ferritin (3,240ng/mL). Dexamethasone 8mg/day was administered for three days followed by slow tapering for two weeks, with rapid normalization of calcium levels and reduction of the inflammatory parameters and ACE (84.95U/L). *C. burnetii* serologies were repeated. The diagnosis of acute Q fever was confirmed by elevated titers of IgG phase II (3,200), IgM phase II titer (50), and IgM phase I (100). Doxycycline therapy was continued for a total of 14 days. The patient’s condition improved progressively and normalization of inflammatory parameters, ACE levels, and liver function tests was achieved. After three months, the patient was asymptomatic and the 18-F-FDG PET-CT scan excluded potential vascular or cardiac involvement.

## Discussion

The diagnosis of Q fever is usually laborious due to unspecific and variable clinical manifestations. The diagnosis of Q fever should be considered in patients who have risk factors for the disease, for instance, contact with farm animals, rural living downwind from farms, travel to endemic areas, etc., and signs and symptoms of Q fever infection. The disease is often diagnosed only after other diagnoses have been systematically excluded. Significantly positive serologies may take three to four weeks to become evident and only 39 of 100 people presenting with Q fever are positive in their first test [1,3,4]. As occurred in this case, the diagnosis of primary infection was made by the detection of a four-fold increase in phase II IgG or IgM antibodies between two serum samples taken three to six weeks apart. Generally, titers of phase II IgG of >200 and/or IgM of >50 are considered significant for the diagnosis of primary Q fever infection.

Biological markers of autoimmunity are frequently present in acute Q fever. Anti-smooth muscle antibodies, antineutrophil cytoplasmic antibodies, and antinuclear and antiphospholipid antibodies have all been detected during acute Q fever [4]. The latter, together with lupus anticoagulant, may also be found and are associated with acute Q fever endocarditis and/or the development of persistent infection. In this case, the patient presented a positive ANA and anti-dsDNA at low titers that were interpreted as an epiphenomenon. No systemic symptoms of lupus developed after the treatment of the infection.

The clinical presentation can be very pleomorphic, though acute hepatitis is a common form of presentation of acute Q fever. Three major forms of hepatitis may be encountered: 1) an infectious hepatitis-like form of hepatitis with hepatomegaly but seldom with jaundice, 2) clinically asymptomatic hepatitis, and 3) prolonged fever of unknown origin with characteristic granulomas on liver biopsy.

Bone marrow examination should always be performed as part of the evaluation for fever of unknown origin and, in this case, was fundamental for the diagnosis. Granulomas are frequently found in infectious diseases such as tuberculosis, syphilis, histoplasmosis, and brucellosis. Bone marrow lesions associated with Q fever are rarely reported. Different types of granulomas have been seen in histologic sections of patients with Q fever, ranging from proliferative granulomas and necrotized granuloma to a more specific “doughnut granuloma.” Doughnut granuloma is characterized by a lipid vacuole in the center of the granuloma, rimmed by polymorphonuclear leukocytes and epithelioid cells. This type of granuloma is frequently described but not pathognomonic of Q fever. It has also been described in patients with brucellosis, leishmaniasis, infectious mononucleosis, and cytomegalovirus infection [4-6].

The elevated levels of ACE can be explained by the total body granuloma load. High ACE levels are commonly associated with sarcoidosis. However, they can also be found in other conditions such as tuberculosis and other infectious granulomata, liver disease, lymphoma, diabetes, hyperthyroidism, or even as a benign familial condition [7]. In fact, Q fever should be considered a granulomatous disease. In this case, normalization of ACE levels after Q fever treatment seems to support the association between this biomarker and the granulomatous response during* Coxiella* infection.

Some cases of granulomatous hepatitis have prolonged febrile illness with poor response to antibiotics that seem to result from a persistent immune cell activation. Moderate doses of steroids can be useful in these patients [8].

The prognosis of acute Q fever is generally good. Risk factors for chronicity have been reported: immunosuppression, valvulopathy, pregnancy, and prosthetic joints. Eldin et al. recommend a close serological follow-up of all patients with *C. burnetii* primary infection at three and six months for early detection of an increase in antibody titers and the performance of an 18 F-FDG PET-CT.

## Conclusions

Q fever is a zoonotic disease caused by* C. burnetii *that occurs worldwide and should be considered in the differential diagnosis of FUO. There is considerable scope for misdiagnosis so that a detailed clinical history (including environmental exposures) and a systematic diagnostic approach are crucial. Bone marrow involvement and its clinical consequences are rarely described in the literature. This case highlights a new perspective of Q fever as a systemic granulomatous disease.
